# *Pseudomonas aeruginosa* and the Oropharyngeal Ecosystem of Tube-Fed Patients

**DOI:** 10.3201/eid0908.030054

**Published:** 2003-08

**Authors:** Arthur Leibovitz, Michael Dan, Jonathan Zinger, Yehuda Carmeli, Beni Habot, Rephael Segal

**Affiliations:** * Tel-Aviv University, Sourasky, Israel

**Keywords:** Nasogastric tube feeding, oral flora, *Pseudomonas aeruginosa*, reservoir, biofilm, research

## Abstract

We evaluated whether elderly patients fed with nasogastric tubes (NGT) are predisposed to *Pseudomonas aeruginosa* colonization in the oropharynx. Fifty-three patients on NGT feeding and 50 orally fed controls with similar clinical characteristics were studied. The tongue dorsum was swabbed and cultured. *P. aeruginosa* was isolated in 18 (34%) of the NGT-fed group but in no controls (p<0.001). Other gram-negative bacteria were cultured from 34 (64%) of NGT-fed patients as compared with 4 (8%) of controls (p<0.001). Antibiotic susceptibility of the oropharyngeal *P. aeruginosa* isolates was compared with that of isolates from sputum cultures obtained from our hospital’s bacteriologic laboratory. The oropharyngeal isolates showed a higher rate of resistance; differences were significant for amikacin (p<0.03). Scanning electron microscope studies showed a biofilm containing *P. aeruginosa* organisms. The pulsed-field gel electrophoresis profile of these organisms was similar to that of *P*. *aeruginosa* isolates from the oropharynx. NGT-fed patients may serve as vectors of resistant *P. aeruginosa* strains.

The oral cavity has long been considered a potential reservoir for pathogenic microorganisms. It is the only normally accessible site in the body that has hard, nonshedding surfaces for microbial colonization. Those unique tissues allow the accumulation of extracellular products and the formation of biofilms that serve as culture media for bacteria and contribute to the development of antibiotic resistance (*1*). Within the mouth, distinct habitats provide different ecologic conditions including mucosal surfaces, cheeks, palate, periodontal region, tongue, and abiotic structures (teeth). Ecologic conditions within the mouth may vary and change the ecosystem, facilitating the growth of pathogenic organisms.

Patients on nasogastric tube (NGT) feeding are a growing segment of the frail elderly population. We have recently reported an increased rate of gram-negative bacteria in the nasopharynx of these patients, including a high proportion of *Pseudomonas aeruginosa* (*2*). *P. aeruginosa* has a predilection for wet sites and respiratory equipment and may create reservoirs that threaten hospitalized patients (*3*). The oropharynx of NGT-fed elderly patients may provide such an ecosystem and promote the colonization of *P. aeruginosa.* This colonization could be due to several factors such as the papillary structure of the dorsum of the tongue, the lack of mastication and swallowing (eliminating their mechanical cleansing effect), and the tube itself. *P. aeruginosa,* a well-known biofilm-producing microorganism (*4*,*5*), may be exploiting the NGT to create a thriving habitat.

The purpose of this study was to reconfirm the high incidence of *P. aeruginosa* isolations from the oropharynx of NGT-fed elderly patients, determine its antibiotic susceptibility, and explore the possibility of biofilm formation on the feeding tube. If these assumptions are true, the oropharynx of NGT-fed patients could constitute a potential reservoir for *P. aeruginosa* in long-term-care facilities.

## Methods

This prospective cross-sectional comparative study was conducted in the four skilled nursing wards of a 158-bed geriatric hospital. Skilled nursing wards are licensed for providing care for nursing patients who also have an active disease requiring close medical supervision (e.g., NGT feeding, severe bed sores, advanced cancer, or hemodynamic instability). Eligible for the study were all patients who had been receiving NGT feeding for at least 2 weeks.

The control group comprised matched orally fed patients, with no swallowing disturbances, who resided in the same wards. The orally fed patients received a regular solid hospital diet with occasional supplements. Excluded from both groups were patients who had received any antibiotic treatment up to 2 weeks before the study, patients with advanced cancer, and patients who had received chemotherapy or radiotherapy to the neck. Informed consent was obtained from the patients or their proxies.

Cultures were obtained by applying sterile cotton swabs to the base of the tongue dorsum and rubbing the buccal mucosa. The sample was then placed in transport medium. Samples were taken in the morning, before breakfast and the daily oral cleansing procedure.

Within 1 h of collection, specimens were spread on blood and MacConkey agar plates and were incubated at 35°C for 18 h. Five colonies of each morphotype were selected for identification. Gram-negative bacteria, including *P. aeruginosa,* were identified by using the BBL Crystal ID system (Becton Dickinson, Sparks, MD). Antimicrobial susceptibility testing was performed by the disc-diffusion technique, according to guidelines of the National Committee for Clinical Laboratory Standards (*6*), with OXOID test discs (OXOID, Basingstoke, Hampshire, England).

Routine oral hygiene for the tuboenteral patients was performed by cleansing the oral cavity before meals three times a day with lemon-glycerine wadding sticks impregnated with a solution of glycerine-citric acid, lemon flavoring, and sodium benzoate 0.1% (*7*). NGTs in use in our hospital are made from polyvinyl chloride (Duodenal Levin Tube–Maersk Medical, Lynge, Denmark).

For the biofilm study, samples of the oropharyngeal section of the NGT were fixed overnight with 2.5% glutaraldehyde in 0.1 M cacodylate buffer, pH 7.4, washed with the same buffer, dehydrated in increasing concentrations of ethanol, dried with a critical point drier, and coated with gold (Polaron-sem coating unit E5100, Thermo VG Scientific, Beverly, MA). The outer surface of the samples was examined by a Jeol-840A scanning electron microscope (JEOL USA, Peabody, MA). Biofilm studies were performed on four patients with a oropharyngeal culture that was positive for *P. aeruginosa* 2–4 weeks after the NGT was inserted.

From three NGT-fed patients with isolations of *P. aeruginosa,* samples were taken concomitantly from the oropharynx and the NGT surface for strain typing by pulsed-field gel electrophoresis (PFGE). DNA preparation and cleavage with 20 U of Spel endonuclease (New England Biolabs, Eldan, Rosh Ha’ain, Israel) were performed, as originally described (*8*). Electrophoresis was performed in a 1% agarose gel (BMA Products, Hann Woong Yoo, South Korea) prepared and run in 0.5 x Tris-borate-EDTA buffer on a CHEF-DR III apparatus (Bio-Rad Laboratories Ltd. Rishon Le Zion, Israel). The initial switch time was 0.5 s, the final switch time was 35 s, and the run time was 22 h at 6 V/cm with a temperature of 14°C. Gels were stained in ethidium bromide, de-stained in distilled water, and photographed with a Bio-Rad GelDoc 2000 camera. PFGE DNA patterns were compared and interpreted according to the criteria of Tenover et al. (*9*).

The antibiotic susceptibility of *P. aeruginosa* isolates from the oropharynx was compared to that of *P. aeruginosa* isolates obtained from sputum cultures and recorded in the laboratory logbook in the preceding year. Statistical processing was performed by using SPSS software (SPSS Inc., Chicago, IL). Chi-square test was used for comparative studies; p<0.05 was considered significant.

## Results

The study group consisted of 53 elderly long-term-care residents who had been receiving NGT feeding for an average of 14 ± 17 months. The control group consisted of 50 counterparts receiving oral feeding. No statistically significant differences in demographic and medical characteristics occurred between the study groups (Table).

**Table:** Demographic data of study groups^a^

Data	NGT-fed patients^a^ n=53 (%)	Orally fed patients n=50 (%)
Age	78 ± 9	81 ± 9
Dementia	32 (60)	29 (58)
Stroke	29 (55)	22 (44)
Diabetes mellitus	9 (17)	7 (13)
COPD	8 (15)	5 (9)
Residual teeth	20 (38)	17 (33)
Corticosteroids	6 (11)	4 (8)

^a^NGT, nasogastric tube; COPD, chronic obstructive pulmonary disease.

Gram-negative bacteria, including *P. aeruginosa,* were cultured from the oropharynges of 34 (64%) of the 53 NGT-fed patients. *P. aeruginosa* was isolated in 18 (34%) patients of this group or in 60% of the patients colonized with gram-negative bacteria. In the control group, gram-negative bacteria were isolated in only 4 (8%) patients (p<0.001). *P. aeruginosa* was not isolated in any of the control group samples (p<0.001). Two patients from the NGT-fed group had a mixture of other gram-negative bacteria and *P. aeruginosa*. No association was found between predisposing factors such as age, gender, diabetes mellitus, chronic lung disease, presence of residual teeth, and isolation of *P. aeruginosa*.

Antibiotic susceptibility studies of *P. aeruginosa* isolated from the oropharynx showed that the highest susceptibility rates were registered for tazobactam-piperacillin, with 89% of the isolates being susceptible, followed by ceftazidim (79%) and imipenem, (78%). The *P. aeruginosa* isolates from the oropharynx were less sensitive to most antibiotics than those cultured from sputum; for amikacin, this difference was significant (p<0.03).

**Figure 1:**
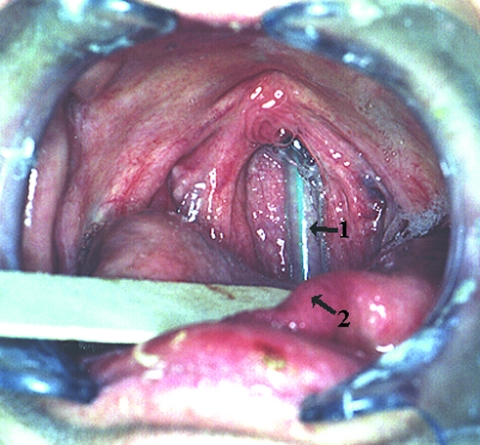
Nasogastric tube embedded in the nasopharynx. 1, nasogastric tube; 2, dorsum of tongue.

**Figure 2:**
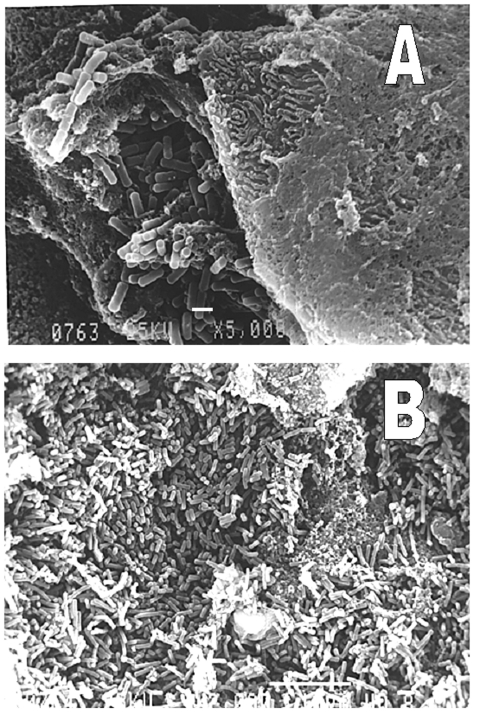
Representative biofilms on nasogastric tubes showing bacterial organisms with typical form of *Pseudomonas aeruginosa.* Scanning electronic microscope. A, scale bar, 1 µm; B, scale bar, 10 µm.

Figure 1 shows the NGT tube embedded in the adjacent anatomical structures. The findings on electron microscopy of an NGT section are shown in Figure 2.

**Figure 3:**
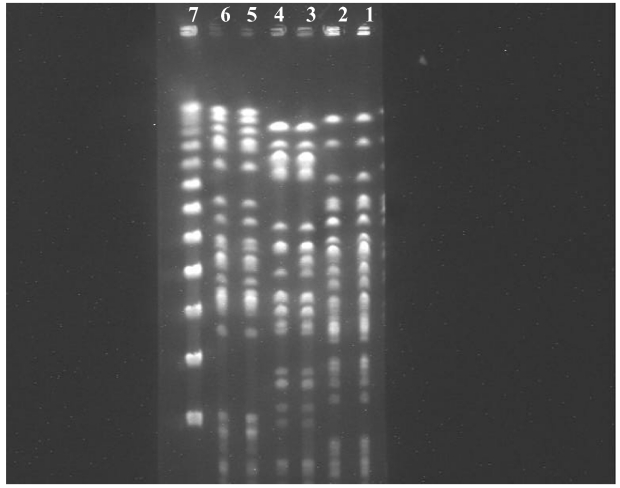
Pulsed-field gel electrophoresis of three pairs of *Pseudomonas aeruginosa* isolates obtained from three patients from nasogastric tubes (lanes 2, 4, 6) and from oropharynx (lanes 3, 5, 7). Lane 1 shows the λ marker size (New England Biolabs, Eldan, Rosh Ha’ain, Israel).

The samples were taken from four patients with cultures positive for *P. aeruginosa,* who had NGTs inserted 2–4 weeks previously. A biofilm with a bacterial organism with a typical form for *P. aeruginosa* was clearly visualized on the outer surface of all four sections. The identity of the bacterium seen in the biofilm was confirmed by culture. Figure 3 shows the results of the PFGE studies on three patients; in each one, the same clone was isolated from both the oral cavity and the NGT.

## Discussion

NGT-fed elderly patients may constitute a human reservoir of *P. aeruginosa.* Determination of the antibiotic susceptibility of these *P. aeruginosa* isolates showed that they had a higher rate of resistance to most antibiotics than to *P. aeruginosa* isolates from sputum obtained from hospitalized patients with bronchopulmonary infections during the same period. This difference reached statistical significance for amikacin. Early studies had reported on the propensity of gram-negative bacteria to colonize elderly patients’ oropharynges (*10*–*13*). Our recently published study (*2*) and the present one are the only reports documenting the colonization of the oropharynx in NGT-fed patients by pathogenic florae.

Two factors may explain the high prevalence of *P. aeruginosa* in the NGT-fed patients. One is the lack of mechanical clearance of the mouth provided by chewing and swallowing, an important mechanism in preventing gram-negative bacteria from colonizing the oropharynx of the elderly (*14*). The other factor is *P. aeruginosa*’s known ability to adhere and form biofilm on plastic tubes, including those made of polyvinyl chloride (*15*,*16*). The biofilms formed on NGTs probably play a major role in the persistence of its colonization of the oropharynges of these patients and interfere with its eradication by antibiotics.

The clinical implications of such a reservoir are far reaching. An opportunistic organism such as *P. aeruginosa* in the oropharynx constitutes a threat to NGT-fed patients, who are at risk for aspiration pneumonia and systemic infections (*17*). Moreover, these elderly NGT-fed patients, most of whom reside in long-term-care facilities, are under “antibiotic pressure” because of frequent clinical infections and may constitute “reservoirs of resistance” (*18*). The existence of a biofilm in such a reservoir would facilitate antibiotic resistance (*19*). A similar effect was reported with polyvinyl chloride endotracheal tubes (*20*). The increased resistance rate of oropharyngeal *P. aeruginosa* isolates to relevant antibiotics in our study as compared with the isolates from sputum is in agreement with this observation.

The epidemiologic importance of a human reservoir of *P. aeruginosa* is not limited to the patient. NGT-fed patients are often transferred to general hospitals and may possibly serve as vectors of resistant organisms to other medical settings (emergency departments, surgical, orthopedic, urologic wards, and intensive care units).

One limitation of our study is that it was performed at a single facility. However, each of the four wards that participated is located in a pavilion separated by 50 m to 100 m from another. Moreover, the factors involved in the oropharyngeal ecosystem colonization by *P. eruginosa* discussed in this study exist everywhere. Similar studies from other long-term-care facilities could provide further evidence.
